# Modulation of Gut Microbiota and Neuroprotective Effect of a Yeast-Enriched Beer

**DOI:** 10.3390/nu14122380

**Published:** 2022-06-08

**Authors:** Valentina Cecarini, Olee Gogoi, Laura Bonfili, Iolanda Veneruso, Giada Pacinelli, Sara De Carlo, Federica Benvenuti, Valeria D’Argenio, Mauro Angeletti, Nazzareno Cannella, Anna Maria Eleuteri

**Affiliations:** 1 School of Biosciences and Veterinary Medicine, University of Camerino, Via Gentile III da Varano, 62032 Camerino, Italy; valentina.cecarini@unicam.it (V.C.); olee.gogoi@unicam.it (O.G.); laura.bonfili@unicam.it (L.B.); mauro.angeletti@unicam.it (M.A.); annamaria.eleuteri@unicam.it (A.M.E.); 2 Department of Molecular Medicine and Medical Biotechnologies, Federico II University, Via Sergio Pansini 5, 80131 Naples, Italy; venerusoi@ceinge.unina.it; 3 CEINGE-Biotecnologie Avanzate, Via G. Salvatore 486, 80145 Naples, Italy; dargenio@ceinge.unina.it; 4 Pharmacology Unit, School of Pharmacy, University of Camerino, Via Madonna delle Carceri 9, 62032 Camerino, Italy; giada.pacinelli@unicam.it (G.P.); sara.decarlo@unicam.it (S.D.C.); federica.benvenuti@unicam.it (F.B.); 5 Department of Human Sciences and Quality of Life Promotion, San Raffaele Open University, Via di Val Cannuta 247, 00166 Roma, Italy

**Keywords:** beer, Alzheimer’s disease, amyloid, inflammation, microbiota

## Abstract

Beer is the most consumed alcoholic beverage worldwide. It is rich in nutrients, and with its microbial component it could play a role in gut microbiota modulation. Conflicting data are currently available regarding the consequences of alcohol and alcohol-containing beverages on dementia and age-associated disorders including Alzheimer’s disease (AD), a neurodegeneration characterized by protein aggregation, inflammatory processes and alterations of components of the gut–brain axis. The effects of an unfiltered and unpasteurized craft beer on AD molecular hallmarks, levels of gut hormones and composition of micro/mycobiota were dissected using 3xTg-AD mice. In addition, to better assess the role of yeasts, beer was enriched with the same *Saccharomyces cerevisiae* strain used for brewing. The treatment with the yeast-enriched beer ameliorated cognition and favored the reduction of Aβ(1-42) and pro-inflammatory molecules, also contributing to an increase in the concentration of anti-inflammatory cytokines. A significant improvement in the richness and presence of beneficial taxa in the gut bacterial population of the 3xTg-AD animals was observed. In addition, the fungal order, *Sordariomycetes*, associated with gut inflammatory conditions, noticeably decreased with beer treatments. These data demonstrate, for the first time, the beneficial effects of a yeast-enriched beer on AD signs, suggesting gut microbiota modulation as a mechanism of action.

## 1. Introduction

Alzheimer’s disease (AD) is a progressive neurodegenerative disease associated with memory impairment and cognitive decline and is the most common cause of dementia in the elderly. The brain regions mainly affected by the disorder are the hippocampus and cerebral cortex. These areas are interested by extensive deposition of protein aggregates, mainly extracellular amyloid-beta (Aβ) plaques and intracellular neurofibrillary tangles of the hyperphosphorylated form of the tau protein. The Aβ(1-40) and Aβ(1-42) peptides are principal components of plaques, and they are the product of the amyloidogenic processing of the amyloid precursor protein by the β- and γ-secretases [1]. Furthermore, dysfunctional proteolytic systems and high levels of both oxidative stress and inflammation characterize the AD brain [2,3]. The inflammatory response initiates with the activation of microglia and the recruitment of astrocytes that release cytokines and other neurotoxic products that contribute to neuronal degeneration and cell death [4]. No definitive drugs are available for this condition and numerous efforts are directed toward the development of new therapeutic approaches able to prevent/ameliorate symptoms as well as to delay the onset of the disorder. Recently, an increasing number of studies are focusing attention on the effects of alcohol and alcoholic beverages on dementia and age-associated disorders including AD. However, conflicting data exist on this topic. In fact, several data reported that alcohol intake can be detrimental and can contribute to cognitive alterations thus increasing the risk of developing neurodegenerative disorders, mainly through induction of oxidative stress, glutamate-associated excitotoxicity and neuronal apoptosis [5]. On the contrary, other findings demonstrate that light to moderate alcohol consumption may have beneficial effects, reducing the risk of developing neurodegeneration [5]. Alcohol’s neuroprotective effect depends upon several factors including the amount of intake and type of beverage consumed [5]. In this regard, alcoholic beverages that contain a reduced concentration of ethanol, such as beer, when taken in low or moderate amounts can help reduce the risk of developing AD [6,7], but the exact molecular mechanisms involved are still unclear. Beer is the most widely consumed alcoholic beverage and is extremely rich in nutrients and micronutrients. Beer’s alcoholic content can range approximately from 0 to 15% *w*/*v*. Essential ingredients for brewing beer are barley, hops, water and yeasts, specifically *Saccharomyces cerevisiae*. Beer composition can vary from one type to another, and among the high number of nutrients, carbohydrates, protein/amino acids, minerals, vitamins and other compounds, such as polyphenols, are the most abundant [8]. Few data are currently available on the neuroprotective properties of beer. Previous findings on human postmortem samples demonstrated that moderate beer consumption, but not wine or spirits, reduced the prevalence of Aβ aggregation in the brain [9].

Furthermore, in addition to being an alcoholic beverage, similar to other fermented food and due to the fact of its microbial component, beer could have probiotic effects on gastrointestinal microbiota, a key component of the gut–brain axis, thus contributing to the maintenance of adequate cognitive and neurological functions. In fact, an increasing number of reports, including preclinical and clinical studies, are now suggesting that a proper modulation of gut microbiota by means of probiotics can ameliorate an AD condition, reducing the cognitive, physiological and neuroanatomical impairment and ameliorating the brain inflammatory and oxidative status [10,11,12,13,14,15].

The aim of this study was to evaluate if moderate consumption of unpasteurized beer could exert beneficial effects in 3xTg-AD mice, a reliable model of human AD, counteracting the cognitive decline and reducing the levels of major hallmarks of the disorder such as amyloid peptides and inflammatory cytokines. Possible effects on components of the gut-brain axis were also evaluated. Furthermore, in order to better highlight the role of yeasts in the modulation of gut microbiota/mycobiota, mice were also treated with an enriched formulation of the beer containing a higher concentration of the same *Saccharomyces cerevisiae* used for brewing beer.

## 2. Materials and Methods

### 2.1. Reagents and Chemicals

Unfiltered nonpasteurized beer with a 9% alcohol content was purchased from Kukà S.r.L. (Italy). In addition, 95% *v*/*v* alcohol was purchased from Carsetti S.r.L. (Italy) and diluted to 9%. SafAleTM T-58 yeast containing *Saccharomyces cerevisiae* and emulsifier E491 was purchased from Fermentis (Italy). Protease inhibitors tosyl phenylalanyl chloromethyl ketone (TPCK) and 4-(2-aminoethyl) benzenesulfonyl fluoride hydrochloride (AEBSF or Pefabloc) were purchased from Sigma-Aldrich S.r.L. (Milano, Italy). The amyloid beta 40 mouse enzyme-linked immunosorbent assay (ELISA) kit and amyloid beta 42 mouse ELISA kit for Aβ(1-40) and Aβ(1-42) peptide determination in brain homogenates were purchased from Invitrogen (Camarillo, CA, USA). The Rat/Mouse Ghrelin (active) ELISA kit, Mouse Leptin ELISA, Rat/Mouse GIP (total) ELISA (Merk EZRMGIP-55K) and the multi-species GLP-1 Total ELISA (Merk EZGLP1T-36K) were bought from Merk group.

### 2.2. Animal Model

AD triple-transgenic mice, B6;129-Psen1tm1Mpm Tg (amyloid precursor protein (APP) Swe, tauP301L) 1Lfa/J (named 3xTg-AD), and the wild-type (wt) B6129SF2 mice (separate line) were purchased from the Jackson Laboratory (Bar Harbor, ME, USA). These transgenic mice contain 3 mutations associated with frontotemporal dementia or familial AD (APPSwe, tau MAPT P301L and presenilin-1 M146V). The animals displayed both a plaque and tangle pathology, with Aβ intracellular immunoreactivity detectable at 3 months of age and hyperphosphorylation of tau protein occurring by 12–15 months of age [16]. Experiments were conducted in accordance with the guidelines of the European Communities Council (86/609/ECC) for the care and use of laboratory animals and were approved by the Italian Ministry of Health (protocol: 1D580.28). Mice were housed in plastic cages (Makrolon, Covestro A.G., Filago, Italy) (4 animals per cage) in a temperature-controlled room (21 ± 5 °C) at 60% humidity on a 12 h light/dark reversed cycle (light was switched on at 8:00 p.m.). The mice were maintained on a laboratory diet (Mucedola, Italy) and tap water ad libitum.

### 2.3. Experimental Design

Eight-week-old 3xTg-AD and wt mice (*n* = 40/line, 50% female) were divided into 4 groups and treated for a period of four months as follows: one group received water (*n* = 10), one group received 9% alcohol (*n* = 10), one group received unpasteurized beer (*n* = 10) and one group received unpasteurized beer enriched with yeast (1.2 × 10^11^ CFU) (*n* = 10). This amount of yeast was added to the beer considering that total microbiota was estimated to be ∼10^13^–10^14^ microbial cells [17] and that fungi consisted of nearly 0.1% of the total microbes in the gut [18], thus approximately 10^11^. Yeasts were daily dissolved in beer and given to the animals. Cages were equipped with two bottles, one containing the experimental beverage (i.e., alcohol, unpasteurized beer or yeast enriched beer) and the other containing water. Beverages were replaced every day, once a day, by the operator. The amount of ethanol, beer, yeast enriched beer and water consumed was measured daily by comparing the volumes in the bottles. Preliminary studies housing mice in single cages were performed to ensure that all animals drank the experimental beverages. Mice were monitored for the amount of water or beer consumed for a period of one week. Bottles were weighted twice a day in order to check the volume of the remaining solution. Both wt and 3xTg-AD mice drank approximately 6–7 mL of the experimental drink during the day. The liquid lost during handling by the experimenter or evaporation was estimated including the same sets of bottles on empty cages. During the treatment, body weight was monitored every week to ensure proper food intake. At sacrifice, blood, intestine with feces and brains were collected. Tissues and plasma, promptly treated with protease inhibitors (i.e., Pefabloc and TPCK), were stored at −80 °C.

### 2.4. Preparation of Brain Samples

Hippocampus (HIP) and prefrontal cortex (PFC) were homogenized (1:5 weight/volume of buffer) in 50 mM Tris buffer, 150 mM KCl and 2 mM EDTA, pH 7.5. Homogenates were immediately centrifuged at 13,000 rpm for 20 min at 4 °C, and an aliquot of the supernatant was used for Western blotting and other biochemical tests, whereas another aliquot was immediately supplemented with protease inhibitors (i.e., Pefabloc and TPCK) for ELISA determinations. The Bradford method was used to measure the protein concentration in homogenates using bovine serum albumin (BSA) as a standard [19].

### 2.5. Preparation of Plasma Samples

Blood samples were collected in tubes with 10% *w*/*v* (g/100 mL) of K_2_-EDTA, centrifuged at 13,000 rpm for 20 min at 4 °C. Plasma was promptly added with proteases inhibitors.

### 2.6. Western Blotting

Brain homogenates (20 μg of proteins) were loaded on 12% SDS-PAGE and electroblotted onto polyvinylidene fluoride (PVDF) membranes (Millipore, Milano, Italy). Membranes were activated with methanol and blocked with 5% BSA in freshly prepared TTBS (Tween 20 plus Tris-HCl and NaCl, pH 7.5). Antibodies were diluted in 2% BSA in TTBS. Proteins were detected with the enhanced chemiluminescence (ECL) system (Amersham Pharmacia Biotech, Milano, Italy) using a ChemiDoc MP system. Primary antibodies (1:500 dilution), used to detect pro- and anti-inflammatory cytokines, were from Abcam plc (Cambridge, UK), whereas secondary antibodies were obtained from Santa Cruz Biotechnology (Heidelberg, Germany, 1:500 dilution). Molecular weight markers (6.5–205 kDa) were included in each gel. Glyceraldehydes-3-phosphate dehydrogenase (GAPDH) was used as a control to check equal protein loading (1:500 dilution). Membranes were stripped using a stripping buffer containing 200 mM glycine, 0.1% SDS and 1% Tween 20. Immunoblot images were quantified using ImageJ 1.52p software (NIH, Bethesda, MD, USA).

### 2.7. Behavioral Test

The novel-object recognition (NOR) test was used to evaluate mice memory integrity. Experimental procedures were performed during the dark phase of the light/dark cycle, from 8:00 a.m. to 3:00 p.m., by investigators blind to the experimental conditions as previously described [20]. Before the test, animals were handled for three days to accustom them to the experimenter. The NOR test was conducted over two days. The first day mice were allowed to explore the empty arena for 5 min to acclimate them to the experimental environment. The second day comprised two 10 min trials spaced 3 h apart. During the first trial (familiarization phase), mice were allowed to explore two identical (familiar) objects. During the second trial (test phase), mice were allowed to explore one familiar and one novel object. The time the rodent spent exploring each object during the test trial provided a measurement of memory integrity, as animals are expected to spend more time exploring the novel object. Objects were different in shape, color and texture and maintained throughout the study to obtain reproducible data. Preliminary experiments were conducted to verify that selected objects elicited the same amount of spontaneous investigation. The results are expressed as the NOR discrimination index (the ratio between the time spent exploring the novel object and the total time spent exploring both objects during the test trial).

### 2.8. ELISA for Aβ Levels Determination

HIP and PFC of the control and treated mice were assayed using ELISA to measure Aβ(1-40) and Aβ(1-42) levels. Based on preliminary tests, samples were diluted at 1:5 with diluent buffer provided with the ELISA kits. Plates were read at 450 nm on a visible plate reader (Biotrak, Amersham). Assays were performed according to the manufacturer’s directions.

### 2.9. ELISA for Hormones Ghrelin, Leptin and GIP, and GLP-1

Plasma hormone concentrations were measured through sandwich ELISA using plasma treated with protease inhibitors (i.e., Pefabloc and TPCK). Plates were read at 450 nm on a visible plate reader, and the values were corrected from the absorbance at 590 nm after acidification of the formed products.

### 2.10. ELISA for Cytokines

The HIP, PFC, and plasma samples, from the wt and 3xTg-AD mice, added with protease inhibitors were also used to measure pro- and anti-inflammatory cytokines using the following ELISA kits: the IL-10 Mouse ELISA Kit, the IL-1β Mouse ELISA Kit, the TNF-α Mouse ELISA Kit, High Sensitivity and the IL-4 Mouse ELISA Kit (Thermo Fisher Scientific Inc., Waltham, MA, USA), following the manufacturer’s instructions.

### 2.11. Microbiota and Mycobiota Analyses

As specified above, fecal samples from the wt and 3xTg-AD mice were collected at the time of sacrifice, immediately cooled on dry ice and stored at −80 °C until analysis. Genomic DNA was extracted using the 16 LEV Blood DNA kit and the Maxwell 16 instrument (both from Promega, Madison, WI, USA) as previously reported [21]. Two blank samples were also collected as the control of this analytical step to check for any environmental contamination occurring during the DNA extraction procedure. To deeply investigate the microbiome composition of all the collected samples, both bacterial and fungal communities were analyzed. For bacteria analysis, a 500 bp amplicon, covering the V4-V6 hyper-variable regions of the 16S rRNA gene, was obtained as previously described [22]. Then, a second-round PCR was performed to univocally tag different samples allowing for their multiplexing. In each PCR step, 2 negative controls were included to be further processed as contamination controls of the whole analytic procedure. The obtained multiple amplicon libraries were quality assessed (TapeStation, Agilent Technologies, Santa Clara, CA, USA) and quantified (Qubit, Thermo Fisher Scientific, Waltham, MA, USA) before being sequenced with the V3 300X2 PE MiSeq protocol (Illumina, San Diego, CA, USA), according to the specifications of the manufacturer. For fungi analysis, specific primers were used for ITS1 amplification [23]. After the first-round PCR to specifically amplify the target region, the amplicons were treated as specified above for the 16S rRNA amplicon. In addition, in this case, PCR controls were processed together with the samples to provide analytic controls for any environmental contaminant.

The FASTQ files were sent to the CRG bioinformatic facility (https://biocore.crg.eu/wiki/Main_Page, accessed on 28 October 2020) for primary data analysis. After an initial quality check with FastQC [24], sequences were processed using the mothur tool (version 1.44.1) [25], following the workflow described on the authors’ website (https://mothur.org/wiki/miseq_sop/, accessed on 3 December 2020). Reference sequences for the bacterial 16S rRNA data analysis were obtained from the SILVA database, version 138 [26], and used for mapping the data and grouping the reads into operational taxonomic units (OTUs) in the mothur framework. Reference sequences for ITS data analysis were obtained from the UNITE database, version 4 February 2020 [27]. Secondary analysis of the metagenomic data was performed using the R packages “Phyloseq” v.1.30.0 [28] and “microbiome” v.1.8.0 [29] to include the estimation of alpha- and beta-diversity [30], and the identification of significantly enriched taxa in studied groups, using the R package “DESeq2” v.1.26.0 [31]. Moreover, the mothur output package was used for further analyses using the Microbiome Analyst tool [32]. Samples richness and/or evenness were evaluated, and the ANOVA test was performed to assess significant differences. Unweighted and weighted Unifrac distance measures were used to evaluate beta diversity coupled with the PERMANOVA test to verify the significance of the samples grouping. Differential abundance analysis was carried out using univariate statistical comparisons based on parametric tests (i.e., *t*-test/ANOVA); *p*-values were adjusted using the FDR method.

### 2.12. Statistical Analyses

Data presented in histograms are expressed as the mean values ± S.D. Statistical analysis was performed using Sigma-stat 3.1 software (SPSS, Chicago, IL, USA). Data were analyzed by one-way ANOVA, followed by the Bonferroni post hoc when appropriate. Wt and 3xTg-AD mice were analyzed separately. Statistical significance was set to the conventional *p* < 0.05.

## 3. Results

### 3.1. Effect of Beer Consumption on Cognitive Performance

**Figure 1:**
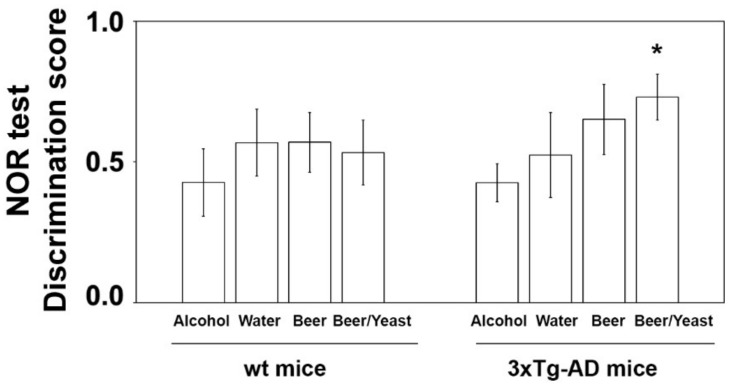
Effect of treatment on the NOR discrimination index in wt and 3xTg-AD mice. Treatment with alcohol, beer or beer/yeast did not affect discrimination index in wt mice, whereas yeast enriched beer (beer/yeast) significantly increased the NOR discrimination index in 3xTg-AD mice. Statistical significance: * *p* < 0.05 vs. water group.

The effect of the treatments was first evaluated on the consolidation process of memory and learning through the novel object recognition (NOR) test [33]. No significant difference was observed in the discrimination scores of wt mice (Figure 1). As for 3xTg-AD mice, ANOVA found no overall effect of treatments. However, since data observation suggested that beer/yeast and beer treatments showed a better discrimination index than water, and being all independent groups, we also compared these two groups with water by *t*-test. Interestingly, we found that beer/yeast, but not beer alone, showed a discrimination index significantly higher than water-treated 3xTg-AD animals, indicating the beneficial effect of this treatment on hippocampus functions and recognition memory (Figure 1).

### 3.2. Effect of Beer Consumption on Amyloid-β Levels

**Figure 2:**
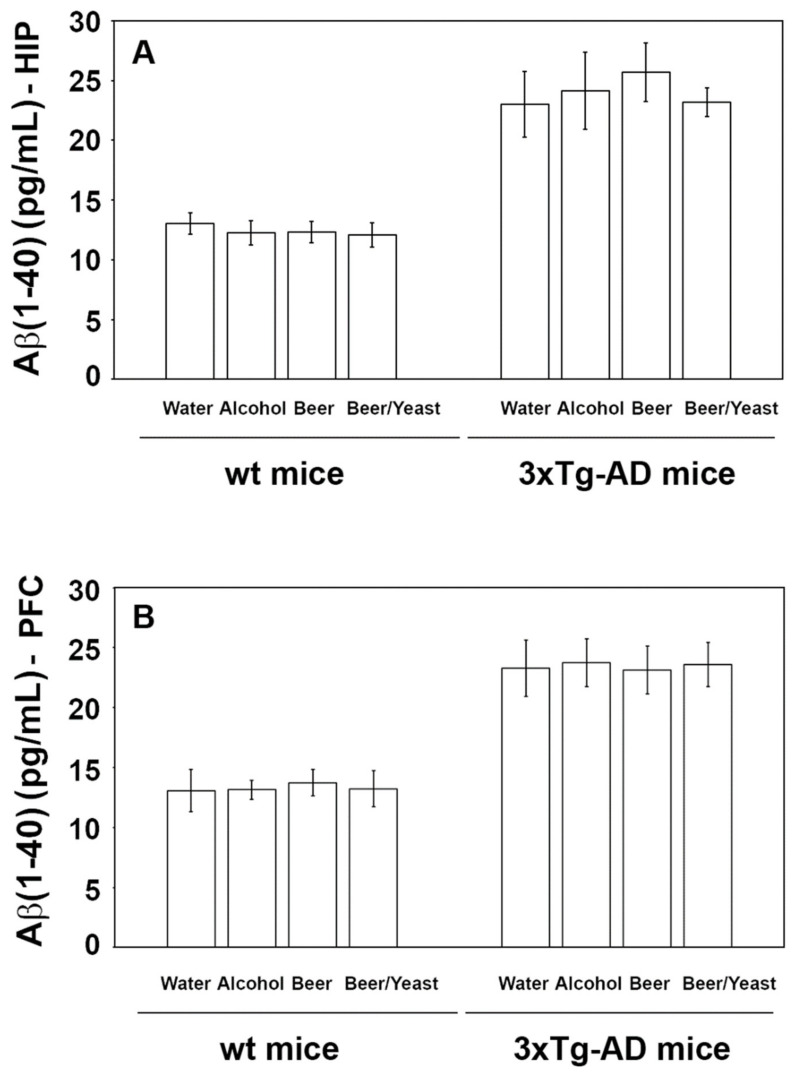
Levels of the Aβ(1-40) peptide measured by ELISA on brain homogenates of the wt and the 3xTg-AD mice treated with water, alcohol, beer and beer/yeast. Treatments did not affect the level of Aβ(1-40) in the HIP (panel **A**) and PFC (panel **B**) of the wt mice and 3xTg-AD mice. Data are expressed as pg/mL of Aβ(1-40).

Accumulation of amyloid beta peptides into plaques is a major hallmark of AD. To evaluate the effect of the treatment on the amount of these proteins, we measured the levels of amyloid (1–40) and (1–42) in the hippocampus and frontal cortex of the control and treated animals. As shown in Figure 2, the treatments were not effective in reducing the levels of the Aβ(1–40) peptide, neither in the wt nor in the 3xTg-AD mice.

**Figure 3:**
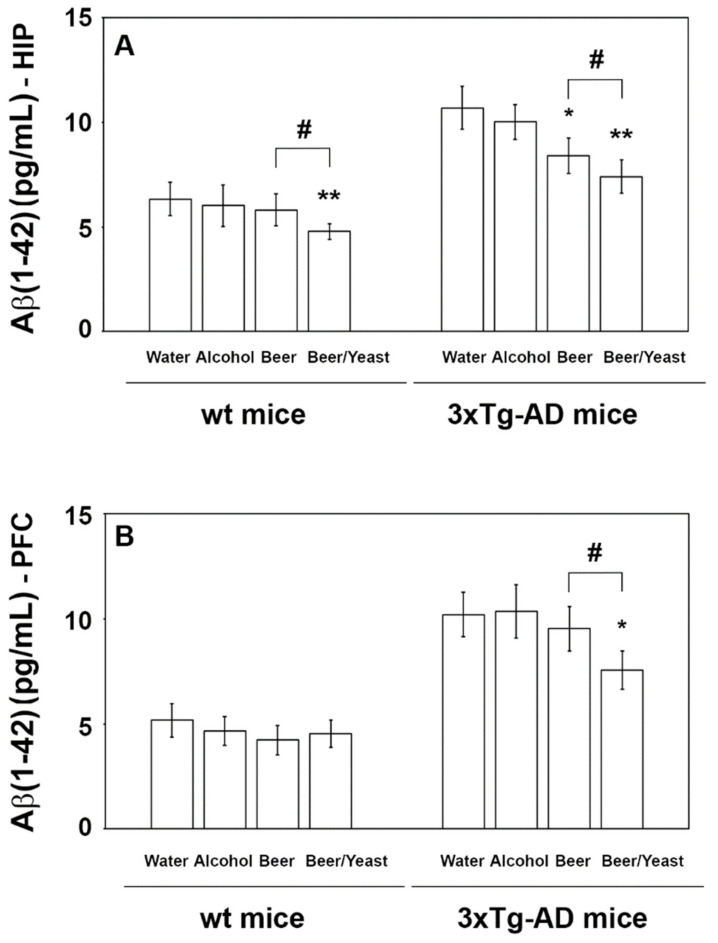
Levels of the Aβ(1-42) peptide measured by ELISA on brain homogenates of the wt and 3xTg-AD mice treated with water, alcohol, beer and beer/yeast. In wt mice, Aβ(1-42) was decreased in the HIP of mice treated with beer/yeast (**A**), but it did not change in the PFC (**B**). Beer and beer/yeast decreased the level of Aβ(1-42) in the HIP of 3xTg-AD mice (**A**). Beer/yeast decreased the level of Aβ(1-42) in the PFC of 3xTg-AD mice (**B**). Concentrations are expressed as pg/mL. (HIP: * *p* < 0.05, B vs. W and A; ** *p* < 0.01, B/Y vs. W and A; # *p* < 0.05, B/Y vs. B; PFC: * *p* < 0.05, B/Y vs. W and A; # *p* < 0.05, B/Y vs. B).

Conversely, regarding the Aβ(1-42) peptide, which is the most toxic and prone to aggregation, post hoc analyses revealed decreased levels in the HIP but not the PFC of the wt mice (Figure 3, panels A and B), whereas both the HIP and PFC of the 3xTg-AD mice showed significantly reduced amounts of this peptide (Figure 3, panels A and B). In detail, if compared to water, Aβ(1-42) in the HIP of the beer and beer/yeast 3xTg-AD groups showed, respectively, a 22 and 30% reduction (Figure 3, panel A) and in the PFC of the beer/yeast 3xTg-AD group a 20% reduction (Figure 3, panel B). These data globally suggest the ability of both beer treatments to act against one of the major hallmarks of AD pathology.

### 3.3. Effects of Beer Consumption on Cytokines Levels

**Figure 4:**
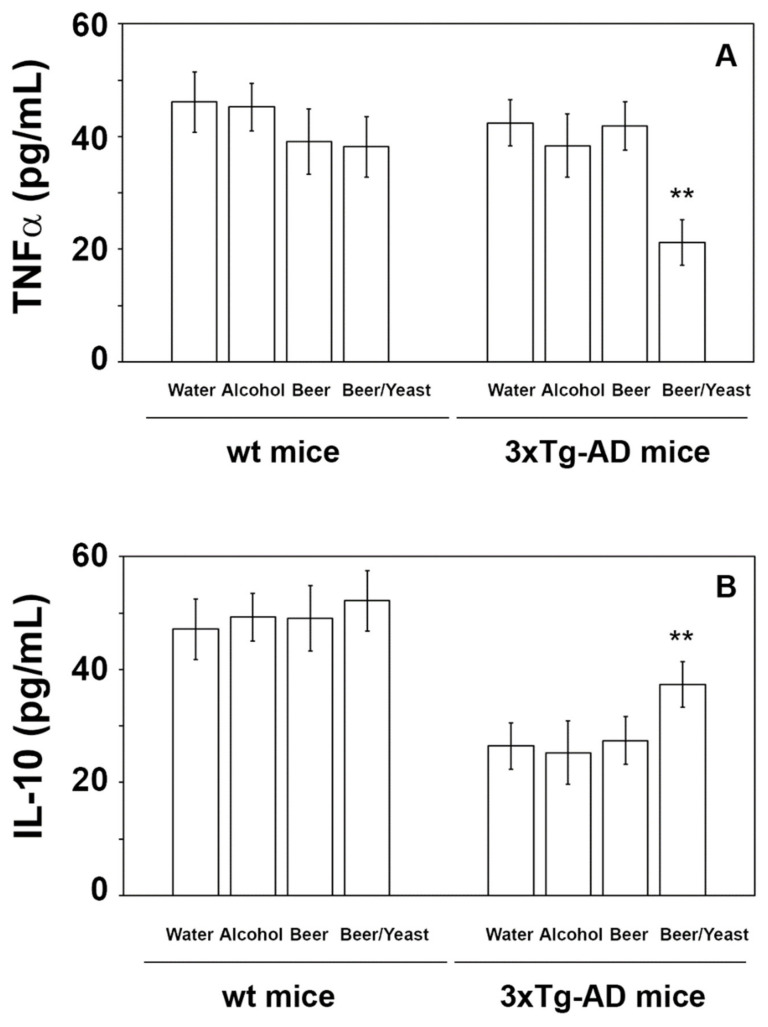
Levels of TNF-α (**A**) and IL-10 (**B**) measured by ELISA on plasma samples of the wt and 3xTg-AD mice treated with water, alcohol, beer and beer/yeast. No changes were detected in the wt animals, whereas TNF-α decreased and IL-10 increased in 3xTg-AD mice upon beer/yeast administration. Concentrations are expressed as pg/mL. HIP: hippocampus; PFC: prefrontal cortex. (** *p* < 0.01 B/Y vs. W, A and B).

**Figure 5:**
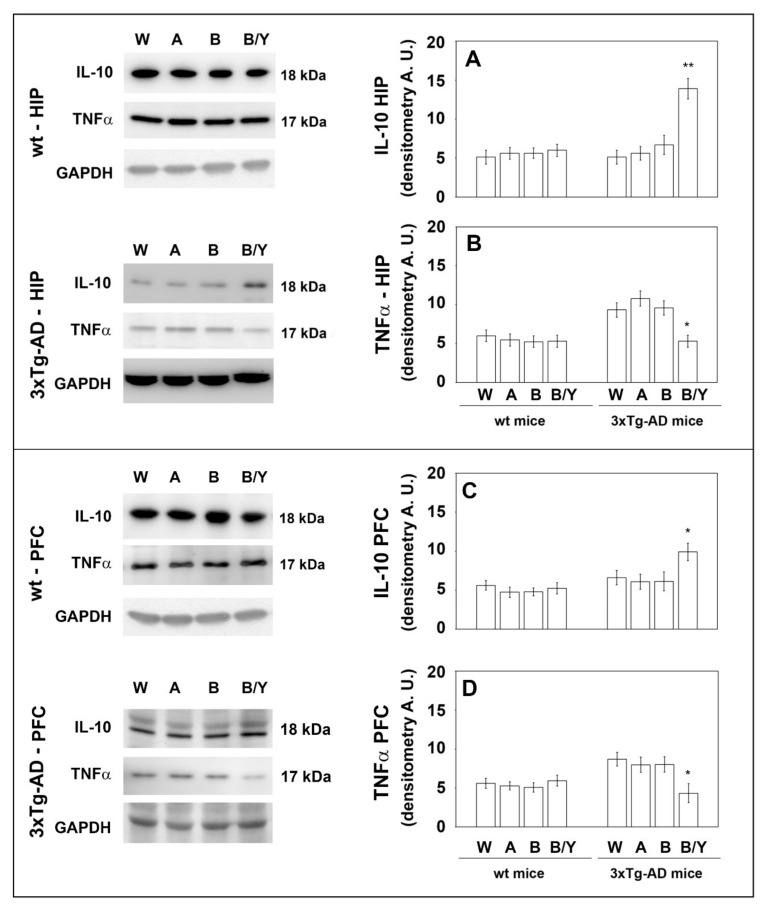
Levels of TNF-α and IL-10 measured by WB on brain samples of the wt and 3xTg-AD mice treated with water, alcohol, beer and beer/yeast. IL-10 expression increased in the HIP and PFC of 3xTg-AD mice (panels **A**–**C**), whereas TNF-α expression decreased in the tested brain regions (panels **B**–**D**). Representative immunoblots and densitometric analyses are shown (A.U.: arbitrary units). Equal protein loading was verified using an anti-GAPDH antibody. HIP: hippocampus; PFC: prefrontal cortex. Data points marked with an asterisk were statistically significant compared to the respective untreated cell line (* *p* < 0.05; ** *p* < 0.01, B/Y vs. W, A and B).

**Figure 6:**
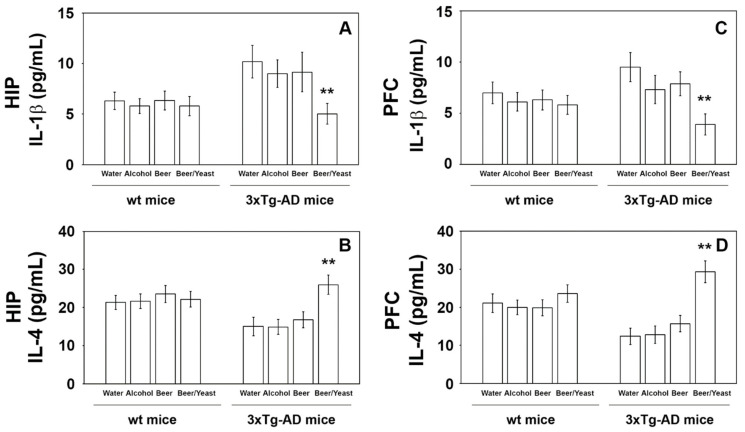
Levels of IL-1β (**A**–**C**) and IL-4 (**B**–**D**) measured by ELISA on brain homogenates of the wt and 3xTg-AD mice treated with water, alcohol, beer and beer/yeast. IL-1β and IL-4 showed, respectively, a decreased and an increased concentration in the HIP and PFC of B/Y-treated 3xTg-AD mice. Concentrations are expressed as pg/mL. HIP: hippocampus; PFC: prefrontal cortex. (** *p* < 0.01, B/Y vs. W, A and B).

Extensive inflammatory processes characterize the AD brain with increased amounts of pro-inflammatory molecules and decreased levels of anti-inflammatory cytokines [34]. To evaluate the possible effects of beer consumption on the inflammatory status of control and treated animals, both wt and transgenic mice, we measured the amounts of pro- (IL-1β and TNF-α) and anti-inflammatory (IL-4 and IL-10) cytokines in both plasma and brain using ELISA kits and WB assays. Samples from the control and treated wt mice showed no difference in the levels of the cytokines TNF-α and IL-10 (measured in the plasma (Figure 4) and in the brain (Figure 5)) and IL-1β and IL-4 (measured in the brain (Figure 6)). Conversely, an evident modulation of the inflammatory condition was obtained in the 3xTg-AD mice treated with the yeast-enriched beer formulation. In detail, comparing this group with water, the pro-inflammatory molecule TNF-α showed a 50% decrease in the plasma (Figure 4, panel A) and in both brain regions of the beer/yeast treated mice (Figure 5, panels B and D). IL-1β significantly decreased in the HIP (50% decrease) and PFC (60% decrease) of mice treated with beer/yeast (Figure 6, panels A and C). In the same samples, an evident increase was observed for the anti-inflammatory molecules IL-10 and IL-4. As for IL-10, the most evident increase was observed in the HIP of beer/yeast-treated mice (2.7-fold increase compared to the water group) (Figure 5, panel A). Finally, IL-4 showed a 1.73- and 2.36-fold increase, respectively, in the HIP and PFC of beer/yeast-treated mice compared to the water-treated animals (Figure 6, panels B and D).

### 3.4. Effect of Beer Consumption on Gut Hormones Levels

We then explored components of the gut–brain axis in order to assess its involvement in the obtained results. The effect of the treatment was evaluated on the concentration of gut hormones, such as leptin, ghrelin, GIP, and GLP-1, determined in plasma samples using ELISA kits. The results showed that treatment with beer and yeast-enriched beer did not significantly alter the levels of the four tested hormones compared to controls (Figure S1). In line with these findings, the body weight of treated mice showed no alterations during the treatment period with respect to the controls (Figure S2).

### 3.5. Bacterial Communities’ Evaluation through 16S rRNA Analysis

An average of 29,030 reads/sample were obtained, allowing the identification of 126 different OTUs. The six negative controls (i.e., blanks), used to exclude any environmental contamination during DNA extraction and PCR amplifications, gave no reads and, thus, were removed from the data analyses. Considering that the gut microbiota of AD patients and animal models displays reduced diversity and a typical taxonomic composition compared to the microbiota of healthy controls [10,35], the presence of bacterial dysbiosis in the 3xTg-AD mice was verified, and the ability of treatments to promote the establishment of beneficial taxa was studied. In particular, since our data showed that in 3xTg-AD mice both beer and beer/yeast consumption were able to significantly modify Aβ(1-42) peptide and cytokines expression, we evaluated the effects of these two treatments on the AD microbiota composition.

**Figure 7:**
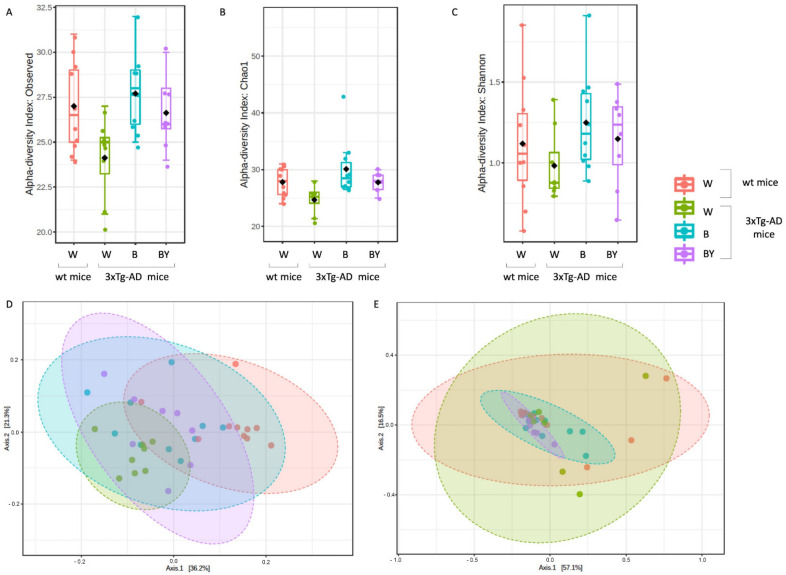
Alpha and beta diversity of the bacterial communities identified for each treatment in the wt (W) and 3xTg-AD mice (W, B and B/Y). Alpha diversity was measured using different metrics, observed species (*p* = 0.02, ANOVA, panel **A**), Chao 1 (*p* = 0.02, ANOVA, **B**) and Shannon index (*p* = 0.36, ANOVA, **C**), to evaluate the within-sample diversity and assess both the richness and evenness of each study group. Taken together, the plots show that the 3xTg-AD mice administered with water had a lower richness and evenness with respect to the wt mice, and that the treatments were able to positively affect the bacterial communities’ heterogeneity. Beta diversity was also evaluated to check between-group diversity. Unweighted (panel **D**) and weighted (panel **E**) Unifrac distances were measured. Statistical significance was measured by PERMANOVA test (*p* < 0.001 and *p* < 0.21, respectively).

**Figure 8:**
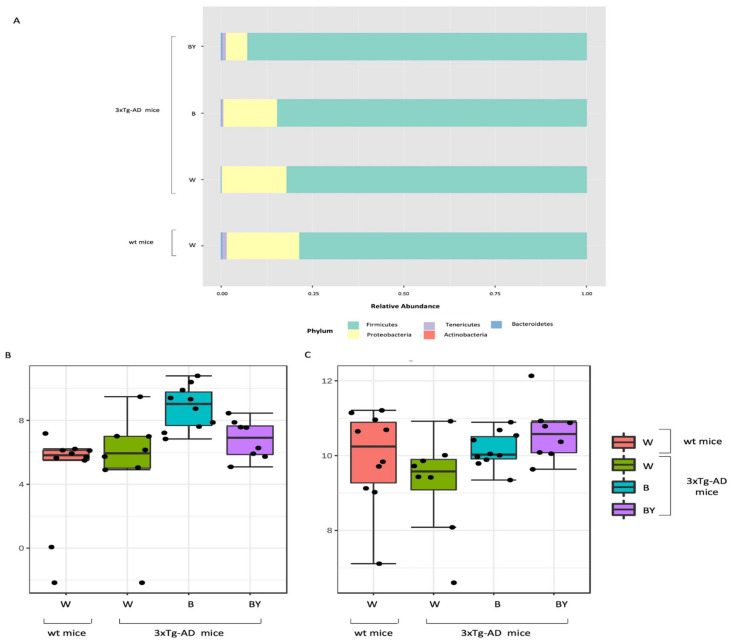
Taxonomic assignment of the gut bacterial communities. Phylum-level taxonomic assignment highlights a different microbial composition between the study groups. According to treatment, it is possible to observe an increase in Firmicutes and a reduction in Proteobacteria (**A**). Classical univariate analysis (i.e., *t*-test/ANOVA) was used to highlight significantly different taxa; at genus level, the genus *Bilophila* was increased in both the treated 3xTg-AD mice (**B**), while *Ruminococcaceae_unclassified* abundance seemed to be restored by the treatments, especially by beer/yeast (BY) administration (**C**).

Alpha diversity was measured to evaluate two key components: richness and evenness. Interestingly, the 3xTg-AD mice administered with water showed both a reduced richness (Figure 7, panels A and B) and evenness (Figure 8, panel C) with respect to the wt mice as assessed by the observed species, Chao 1 and Shannon indices. Moreover, within the 3xTg-AD mice groups, beer and beer/yeast treatments were able to affect both richness and evenness, which appeared to be restored at levels more similar to the wt mice (Figure 7, panels A, B and C). These results indicate that AD is associated to a reduced bacterial abundance and heterogeneity, and these features are improved at all taxonomic levels upon beer consumption. 

To assess the presence of a different bacterial composition between the tested study groups, beta diversity analysis was also evaluated using both the unweighted (Figure 7, panel D, PERMANOVA, *p* < 0.001) and weighted (Figure 7, panel E, PERMANOVA, *p* < 0.21) Unifrac distance measures. Since the unweighted Unifrac is a quality-based parameter and the weighted Unifrac is a quantitative-based one, our data suggest that the differences between the compared groups are due more to the kind of taxa, rather than their relative abundances.

Taxonomy assignment showed different bacterial profiles in the individual samples at the phylum level. Merging samples/status, these differences were more evident: in total, five phyla were identified, with *Firmicutes* and *Proteobacteria* being the most abundant in all the studied groups (Figure 8, panel A). In particular, it was possible to observe a reduction of *Proteobacteria* in the beer and beer/yeast treatments in respect to both the wt and 3xTg-AD mice administered with just water. In addition, the two treatments were featured by an increased abundance in both *Tenericutes* and *Actinobacteria* in respect to th eAD-W mice. Finally, the Bacteroidetes phylum appeared less abundant in all the 3xTg-AD mice, irrespective of treatment (Figure 8, panel A).

Thus, to highlight taxa significantly different between the tested conditions, classical univariate analysis (i.e., *t*-test/ANOVA) was performed. Interestingly, we found one phylum, two classes, two orders, two families and three genera significantly different (adjusted *p*-value < 0.05) among the four tested groups. All the significantly expressed taxa are listed in Table S1 (Supplementary Materials). These data confirm a significant reduction in the Bacteroidetes phylum in all the 3xTg-AD mice in respect to the wt; this difference was also present at the class (*Bacteroidia*), order (*Bacteroidales*), family (*Prevotellaceae*) and genus (*Prevotellaceae_unclassified*) levels. Moreover, we found a significant increase of the genus *Bilophila* (*Desulfovibrionaceae* family, *Desulfovibrionales* order, and *Deltaproteobacteria* class) in the B and B/Y groups of transgenic mice (Table S1, Supplementary Materials; Figure 8, panel B). Interestingly, the genus, *Ruminococcaceae_unclassified* (Firmicutes phylum), was reduced in the 3xTg-AD mice administered with water with respect to the wt, but their abundance was increased by both treatments, with a higher effect in the beer/yeast group (Figure 8, panel C).

Finally, to identify the taxa most likely to explain the differences between the study groups, linear discriminant analysis (LDA) effect size (LEfSe) was performed. As reported in Table S2 (Supplementary Materials), at the genus level, we found that the genera *Prevotellaceae_unclassified* and *Bilophila* were significantly more and less abundant, respectively, in the wt compared to the 3xTg-AD mice. Interestingly, the treatments seemed to be able to modify specific taxa resembling a relative abundance more similar to the wt mice in respect to the 3xTg-AD mice administered just with water.

### 3.6. Fungal Communities’ Evaluation through ITS1 Analysis

**Figure 9:**
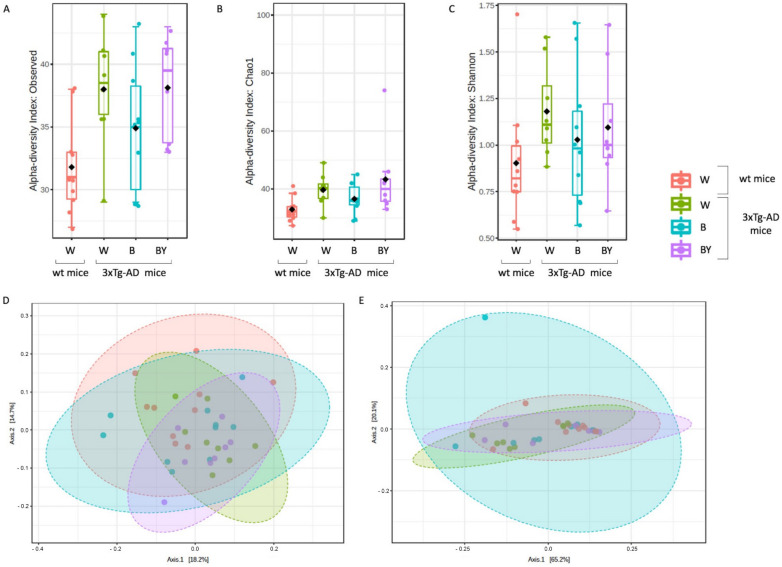
Alpha and beta diversity of the fungal communities identified in the wt and AD-treated and untreated (W, B and beer/yeast (B/Y)) mice. To evaluate the within-sample diversity and assess both the richness and evenness of each study group, the alpha diversity was measured using 3 different metrics, namely, observed species (*p* = 0.01, ANOVA, panel **A**), Chao 1 (*p* = 0.04, ANOVA, panel **B**) and Shannon index (*p* = 0.33, ANOVA panel **C**). 3xTg-AD mice had a higher richness and evenness with respect to the wt mice, and this seemed to not be affected by treatment. Beta diversity was also measured to evaluate the between-group diversity. Both unweighted (panel **D**) and weighted (panel **E**) Unifrac distances were measured using the PERMANOVA test to assess any statistical significance (*p* = 0.028 and *p* = 0.22, respectively).

Fungal-specific internal transcribed spacer (ITS) amplicon sequencing was performed to investigate associations between the fungal gut microbiota and AD and to evaluate a possible positive effect upon beer consumption. To this aim, we obtained a total of 469 OTUs with an average reads/sample equivalent to 95,184. Alpha and beta diversity were measured to assess the within and between groups variability of the identified fungal communities. In particular, observed species, Chao1 and Shannon diversity indices were evaluated to measure both richness and evenness within the tested groups. As shown in Figure 9, the 3xTg-AD mice had a significantly higher richness (panels A and B) and an increased, even if not significant, evenness (panel C). Interestingly, this feature seemed to be irrespective of treatment.

Then, beta diversity was measured as unweighted and weighted Unifrac distances (PERMANOVA test). The unweighted analysis showed a significant difference between the fungal communities of the compared conditions (Figure 9, panel D), not confirmed by the weighted test (Figure 9, panel E) as in the case of the bacterial communities.

**Figure 10:**
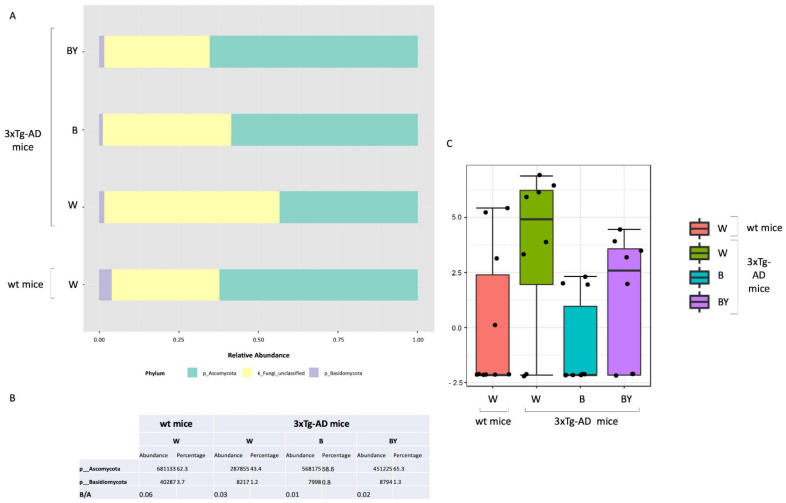
Fungal profiles at the phylum level obtained with the phylogeny-based taxonomy assignment approach. The identified phyla are reported for each study group (panel **A**). Basidiomycota and Ascomycota phyla reads abundance, percentage and ratio are also reported (panel **B**). The *c_Sordariomycetes_unclassified* order was the only significantly different taxa among the tested conditions (panel **C**).

Taxonomic assignment was then carried out. Despite a large fraction of unclassified OTUs, at the phylum level, the Ascomycota phylum was the most abundant in all of the study groups in respect to Basidiomycota (Figure 10, panels A and B). Interestingly, it is possible to observe in the untreated 3xTg-AD mice a reduction in both the Ascomycota and the Basidiomycota phyla with respect to the wt mice, partially restored by the treatments with a higher effect in the case of beer/yeast administration (Figure 10, panel A). However, the ratio between these two phyla did not seem to be affected (Figure 10, panel B). Thus, classical univariate statistical comparison (i.e., *t*-test/ANOVA) was performed highlighting the order *c_Sordariomycetes_unclassified* as the only differentially expressed taxa between the tested conditions. This order increased in the untreated 3xTg-AD mice, and its abundance was reduced by the beer and beer/yeast treatments (Figure 10, panel C); moreover, the significant differences were present also at the family and genus levels. LEfSe analysis gave no significantly different results.

## 4. Discussion

Beer is the most widely consumed fermented beverage in the world, produced from water, malt, hops and yeast, specifically *Saccharomyces cerevisiae* [36]. Emerging studies are now highlighting that moderate consumption of beer may be beneficial and favor healthy aging [37]. Aging results from the accumulation of molecular and cellular alterations, leading to a growing risk of developing disorders such as AD, which is characterized by massive deposition of Aβ peptides in senile plaques and other aggregates that lead to progressive cognitive dysfunctions [38]. Although no definitive treatment exists for AD, a proper modulation of gut microbiota composition is emerging as an effective strategy to ameliorate AD pathology [10,11,39]. For this reason, considering the presence of yeasts and of other microbes or probiotics in fermented beverages, it is reasonable to hypothesize that they could exert a protective effect through an action on gut microbiota. The present work investigated in wt and 3xTg-AD mice the potential beneficial effects of a four-month treatment with an unpasteurized beer, evaluating amyloid-β peptides amounts and inflammatory markers. In addition, shifts in gut microbes’ population, both bacteria and fungi, were detected. The same beer used for the treatment was enriched with the yeast used for brewing beer to better elucidate the role of the microorganisms in the final effect. 

Firstly, the mice’s cognitive performances were analyzed with the NOR test. Discrimination indexes indicated that the treatment with the yeast-enriched beer positively affected the 3xTg-AD mice’s cognitive functions. No effect on behavior was observed in the wt animals.

The observed beneficial effect on behavioral performance on short-term memory prompted us to focus on two important AD molecular hallmarks: the amount of amyloid peptides and the inflammatory condition. In agreement with our working hypothesis and in line with data from behavioral tests, biochemical results showed that the treatment with the yeast-enriched beer was more effective compared to beer alone, indicating the important contribution of the beer yeasts to the observed final effects. In detail, beer treatments successfully diminished the levels of the Aβ(1-42) peptide in the brain of treated AD animals and the addition of the yeast visibly strengthened the final effect, with an evident reduction in the peptide not only in the hippocampus but also in the cortex region of 3xTg-AD mice brain. Conversely, no changes in the Aβ(1-40) amyloid peptide amounts were detected in both the wt and 3xtg-AD mice. These results are in line with a post-mortem study performed by Kok et al. that investigated the association between the consumption of different alcoholic beverages and Aβ pathology, suggesting that beer intake may protect against Aβ aggregation in the brain [8]. 

AD is always accompanied by severe inflammation that slowly leads to neuronal death [34]. Moderate consumption of either wine or beer was previously associated with lower levels of systemic inflammatory markers in three different European areas [40]. Additionally, administration of iso-α-acids, bitter components of beer, suppresses neuroinflammation and improved cognitive function in a mouse model of AD [41]. In light of this evidence, we analyzed plasma and brain levels of pro-inflammatory (i.e., IL-1β and TNF-α) and anti-inflammatory (i.e., IL-4 and IL-10) cytokines, determining that the yeast-enriched beer stimulated a significant anti-inflammatory response in the 3xTg-AD mice. Conversely, treatment with beer did not significantly alter the plasma levels of the considered cytokines. Again, no effect was detected in the wt animals. These data, therefore, suggest that beer enrichment with the brewing yeast definitely improved beer’s ability to decrease important toxic hallmarks of the pathology, such as the inflammatory status, further confirming previous findings on the beneficial effects of yeasts. In detail, these microorganisms, most of all *Saccharomyces cerevisiae*, were characterized for their probiotic effects and for their ability to favor the bioavailability of nutrients, thus improving the nutritional value of foods [42].

To better understand the mechanisms that promote the decrease in the investigated AD signs, we analyzed some of the components of the gut–brain axis, the intricate bidirectional communication system that integrates brain cognitive centers with intestinal functions through neuro-immuno-endocrine mediators [43]. In this regard, we first explored the levels of the gut hormones ghrelin, leptin, GIP and GLP-1 in mice plasma. However, no significant change was observed comparing the four experimental groups, in both the wt and 3xTg-AD mice. Then, we screened the microbiota composition for changes in the richness, that is, the number of species present in a sample, and in the evenness, the related differences in the abundance of species. Treatments with beer and beer/yeast significantly increased the richness in the gut bacterial population of the 3xTg-AD mice making the microbiota of these animals more similar to that of healthy subjects. Interestingly, the 3xTg-AD mice treated with the yeast-enriched beer showed an increase in *Firmicutes* and a simultaneous decrease in *Proteobacteria*. In light of previous studies demonstrating a reduction in the phylum *Firmicutes* and an enrichment of *Proteobacteria* in AD individuals compared to healthy subjects [44], these data demonstrate the positive impact of the treatment on bacterial population composition, suggesting that the modulation of gut microbiota may contribute to the final effect of the treatment. Interestingly, an increase was observed for the genus Bilophila, an anaerobic and sulfite-reducing bacterium and a member of the gut microbiota [45]. It is able to carry out organosulfonate respiration by using taurine and other sulfite donors for energy conservation and producing hydrogen sulfide. The latter bacterial metabolite has been reported as a risk factor for several diseases [46]. However, it was recently pointed out that hydrogen sulfide may have beneficial effects by acting as an antioxidant, signaling molecules and energy [47].

As for the fungal population, beside the very few data currently available on the entire set of fungal species residing in humans [48], it is now widely demonstrated that these microorganisms can control important processes such as the regulation of the immune response and prevention and treatment of bacterial infections and intestinal complications [48,49]. Nevertheless, the mycobiota is still poorly investigated, the majority of metagenomic studies carried out so far being focused just on the bacterial counterpart. As a consequence, an accurate taxa identification is difficult due to the lack of comprehensive databases for fungal reads alignment and is reflected in the high number of unclassified reads. In our study, although minor changes were observed in this group of microorganisms upon treatments, a relevant and interesting shift was detected in the order *Sordariomycetes*, which increased in the untreated 3xTg-AD mice compared to the wt animals, whereas its abundance was reduced by beer and beer/yeast treatments. This is the first report of a relationship between AD and this fungal taxon that was instead previously associated with dysbiosis detected in a series of gut inflammatory diseases including Crohn’s disease, colorectal cancer, myalgic encephalomyelitis and inflammatory bowel disease [50,51]. Interestingly, beer treatment successfully reduced the amount of these fungi in the gut of 3xTg-AD mice, eventually contributing to a reduction in the gut inflammatory condition.

This study provides supportive evidence for a beneficial role of fermented beverages in neurodegenerative disorders associated with aging. Collectively, our results indicate that a moderate intake of a yeast-enriched beer can successfully counteract AD major hallmarks and associated clinical manifestations.

## Data Availability

Data is contained within the article or Supplementary Materials.

## Supplementary Materials

The following supporting information can be downloaded at: https://www.mdpi.com/article/10.3390/nu14122380/s1, Figure S1: Levels of gut hormones in control and treated mice; Figure S2: Mice body weight was monitored during the entire treatment period every week. The image shows body weight changes for control and treated wt and 3xTg-AD mice; Table S1: Significantly different bacterial taxa; Table S2: Linear discriminant analysis (LDA) effect size (LEfSe) between the differently treated AD and wt mice at the bacterial genus level.

## Abbreviations

AD: Alzheimer’s disease; GIP, gastric inhibitory peptide; GLP, glucagon-like peptide-1; HIP, hippocampus; PFC, prefrontal cortex; NOR, novel object recognition; IL-1β, inteleukin-1β; TNF-α, tumor necrosis factor-α; Il-4, interleukin-4; IL-10, interleukin-10.
