# Supplementary material for: Modulation of Gut Microbiota and Neuroprotective Effect of a Yeast-Enriched Beer
Source: Nutrients. 2022 Jun 8;14(12):2380. doi: 10.3390/nu14122380 (PMC9228237; doi:10.3390/nu14122380)
Supplement: Supplementary file 1 [file nutrients-14-02380-s001.zip › nutrients-1740671-supplementary.pdf]

Supplementary Materials

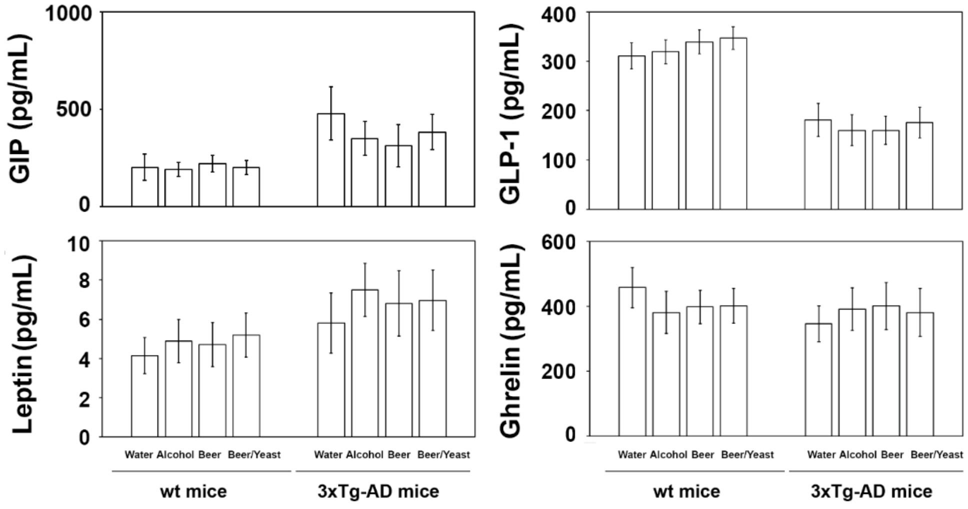

Figure S1. ELISA tests were performed on plasma samples to determine the levels of GIP, Leptin, GLP-1 and Ghrelin in wt and 3xTg-AD mice. The assays were performed following the instructions of the manufacturer (see Materials and Methods for further details).

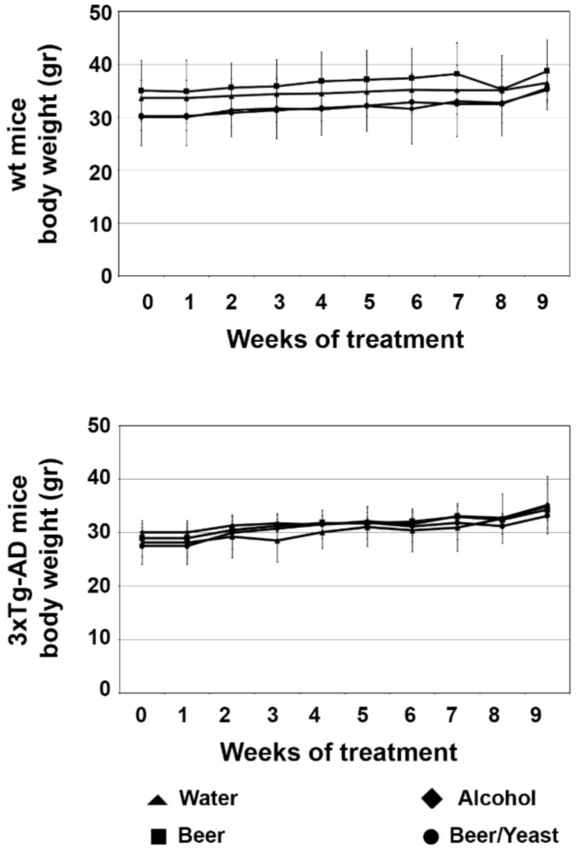

Figure S2. Mice body weight was monitored during the entire treatment period every week. The image shows body weight changes for control and treated wt and 3xTg-AD mice.

Table S1. Significantly different bacterial taxa identified in 3xTg-AD mice by differential abundance analyses as assessed by T-Test/ANOVA (adjusted *p*-value<0.05). Taxa are reported from phylum to genus and ordered based on *p*-values (from the most significant value).

Rank	Taxon	P-value	FDR
Phylum	Bacteroidetes	8.0846E-4	0.0040423
Class	*Bacteroidia*	8.0846E-4	0.0088931
Class	*Deltaproteobacteria*	0.0027272	0.015
Order	*Bacteroidales*	8.0846E-4	0.0097015
Order	*Desulfovibrionales*	0.0027272	0.016363
Family	*Prevotellaceae*	2.1779E-5	4.1381E-4
Family	*Desulfovibrionaceae*	0.0030003	0.028503
Genus	*Prevotellaceae_unclassified*	2.1779E-5	7.1872E-4
Genus	*Bilophila*	0.0010246	0.016905
Genus	*Ruminococcaceae_unclassified*	0.0029797	0.032776

Table S2. Linear Discriminant Analysis (LDA) Effect Size (LEfSe) between the differently treated AD and wt mice at bacterial genus level. LEfSe determines the features most likely to explain differences between groups by coupling standard tests for statistical significance with additional tests assessing the impact of the effect size. Twelve significant genera have been identified and reported herein.

	p-values	FDR	WT_W	AD_W	AD_B	AD_BY	LDA score
**Prevotellaceae_unclassified**	9.7773e-05	0.0032265	4700.8	0	226.39	0	3.37
**Bilophila**	0.00027941	0.0046103	18168	49158	288060	66557	5.13
**Ruminococcus**	0.00066647	0.0073312	157900	0	193670	1997.1	4.99
**Escherichia_Shigella**	0.0014677	0.012108	621560	983160	15491	5638.7	5.69
**Pseudoflavonifractor**	0.0026204	0.017295	4170.8	4849.8	12003	13520	3.67
**Roseburia**	0.0057349	0.027853	17355	156.74	7682.8	35468	4.25
**Ruminococcaceae_unclassified**	0.0059081	0.027853	417220	272810	479490	781440	5.41
**Clostridium_XlVa**	0.01007	0.04154	16051	107800	33003	70799	4.66
**Desulfovibrionales_unclassified**	0.013038	0.047807	10806	0	14317	61268	4.49
**Ureaplasma**	0.018463	0.060926	75388	0	22445	72121	4.58
**Desulfovibrionaceae_unclassified**	0.023302	0.064657	9842	11087	5511.3	70596	4.51
**Coriobacteriaceae_unclassified**	0.023512	0.064657	14291	3527.2	12409	29064	4.11
